# Thrombocytopenia and Anemia After Cardiac Surgery

**DOI:** 10.1002/ajh.27696

**Published:** 2025-05-02

**Authors:** Myriam Beshai, Nour Alhomsi, Theodore E. Warkentin

**Affiliations:** ^1^ Department of Pathology and Molecular Medicine McMaster University Hamilton Ontario Canada; ^2^ Transfusion Medicine Hamilton Regional Laboratory Medicine Program, Hamilton Health Sciences Hamilton Ontario Canada; ^3^ Service of Benign Hematology Hamilton Health Sciences, Hamilton General Hospital Hamilton Ontario Canada

**Keywords:** alloantibodies, anemia, hemolysis, HIT antibodies, thrombocytopenia

## 1 Case Presentation

**An 83‐year‐old Caucasian female with hypertension, dyslipidemia, and diabetes presented to the hospital with acute chest pain, nausea, and diaphoresis; she had a previous history of coronary angioplasty (9 years prior). Acute non‐ST segment elevation myocardial infarction (NSTEMI) was diagnosed based upon non‐specific T wave abnormalities and high‐sensitivity troponin‐I rise (peak, 780 ng/mL; reference range [RR], < 40). She received enteric‐coated aspirin (160 mg first dose; subsequently, 81 mg daily) and 2.5 mg of fondaparinux daily by subcutaneous injection. Heart catheterization, performed with 3000 units (U) of unfractionated heparin [UFH], revealed triple‐vessel coronary artery disease (including 80% stenosis in the left anterior descending artery), with left ventricle function preserved; coronary artery bypass graft (CABG) surgery was scheduled.**

CABG surgery is common in the United States (400 000 procedures per year) [1]. While percutaneous coronary intervention may be used to treat NSTEMI, CABG is often performed for triple‐vessel coronary disease with stenosi(e)s greater than 70%. Patlolla and coworkers [2] reported better long‐term survival in patients undergoing CABG surgery within 7 days following NSTEMI versus CABG surgery performed later. This patient was judged to be a relatively good candidate for CABG. This patient was treated in a Canadian hospital, and thus the antithrombin‐dependent pentasaccharide anticoagulant, fondaparinux—which is approved in Canada for coronary artery thrombosis prophylaxis in STEMI patients based upon efficacy and safety (versus enoxaparin) in this clinical setting [3]—was administered (note: fondaparinux is not approved for coronary thrombosis prophylaxis by the U.S. Food and Drug Administration).

**FIGURE 1:**
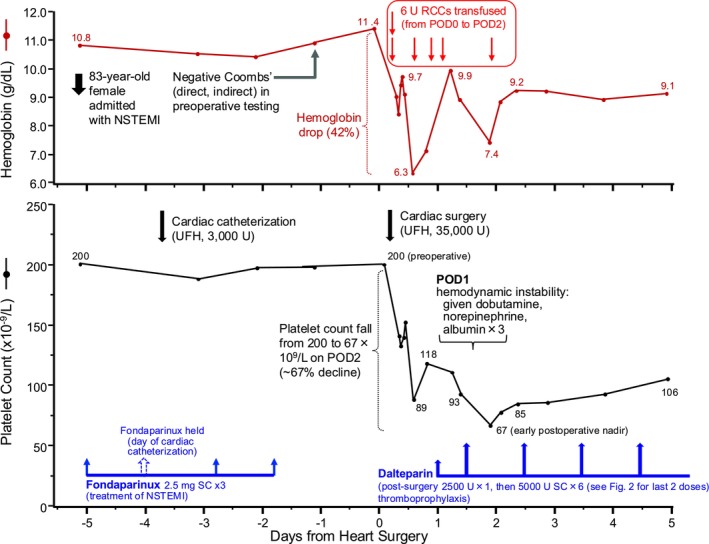
Serial platelet counts (lower panel) and hemoglobin levels (upper panel) 5 days before and 5 days after coronary artery bypass surgery (CABG). The patient received fondaparinux after diagnosis of non‐ST elevation myocardial infarction (NSTEMI). Following CABG, the patient received thromboprophylaxis with low‐molecular‐weight heparin (LMWH), dalteparin. Bleeding following CABG resulted in transfusion of 6 units (U) of red cell concentrates (RCCs), group A RhD+. No red blood cell alloantibodies were detected during routine preoperative screening. CABG, coronary artery bypass grafting; NSTEMI, non‐ST‐elevation myocardial infarction; POD, postoperative day; RCC, red cell concentrate; SC, subcutaneous; U, units; UFH, unfractionated heparin. [Color figure can be viewed at wileyonlinelibrary.com]

**Prior to CABG surgery, the last doses of fondaparinux and aspirin were given 2 and 3 days before surgery, respectively.** **Immediately pre‐surgery, her complete blood count (CBC) showed: platelet count 200 × 10^9^/L (reference range [RR], 150–400), hemoglobin 10.8 g/dL (RR, 11.5–16.5), and white blood cell (WBC) count 8.3 × 10^9^/L (RR, 4.0–11.0), with normal automated WBC differential.** **She was typed as blood group A, RhD‐positive (A+), without red blood cell (RBC) alloantibodies (by indirect Coombs' test); a direct antiglobulin test (DAT, i.e., direct Coombs) was also negative.** **She underwent CABG, with three arteries bypassed, and received a total of 35 000 U of UFH intraoperatively. Her intraoperative and early postoperative course was complicated by bleeding: on the day of surgery, she was transfused a total of 3 units of A+ red cell concentrates (RCCs), and 3 more A+ RCCs during the first two postoperative days.** **Her platelet count dropped by 55% to 89 × 10^9^/L on the day of surgery, and hemoglobin levels dropped by 42% to 6.3 g/dL (nadir) on the day of surgery (Figure** 1 **).**

Bleeding is commonly associated with CABG, as 30% to 50% of patients require a blood transfusion [4]. In part, this reflects the use of UFH as the standard intraoperative anticoagulant, given heparin's effectiveness in preventing device surface‐related thrombosis, simple operating room monitoring (by activated clotting time), short half‐life, non‐hepatic/non‐renal clearance, and reversibility with protamine. Other predispositions to bleeding include reduced platelet number and function, hyperfibrinolysis, and surgical bleeding (e.g., at wound sites) [5]. She received a total of 6 units of RCCs, a relatively large number: in one study [4], only 8% of cardiac surgery patients received more than 4 units of RCCs.

**She received low‐molecular‐weight heparin (LMWH) thromboprophylaxis by standard hospital protocol, namely a first dose of dalteparin (2500 U) administered the day following surgery and 5000 U once‐daily by SC injection thereafter.** **Following her immediate postoperative platelet count fall of approximately 55%, her platelet count declined further; by POD2, her platelet count had fallen by almost 67% (in relation to the preoperative level), accompanied by hemodynamic instability, with lactic acidosis, need for vasopressors, and albumin administration.** **Figure** 1 **summarizes the clinical and laboratory events over the first 10 days (5 days preoperative; 5 days postoperative), including thromboprophylaxis first with fondaparinux (preoperative, for NSTEMI) and later with dalteparin (postoperative); following the initial post‐surgical platelet count decline, there was progressive platelet count recovery from POD2 (nadir) to POD5.**

Despite the aforementioned advantages of UFH anticoagulation during cardiac surgery, one drawback (besides bleeding) is its propensity to cause a potentially severe adverse drug reaction, heparin‐induced thrombocytopenia, or HIT (discussed subsequently). Although both UFH and LMWH are often given following cardiac surgery for routine thromboprophylaxis, LMWH may be associated with a lower “breakthrough” risk of HIT among patients who form HIT antibodies due to intraoperative UFH exposure [6]. At our heart surgery center, LMWH has been the preferred anticoagulant for postoperative thromboprophylaxis since 2012. Although early postcardiac surgery HIT can occur (onset before POD5) as a result of immunization from recent preoperative administration of heparin [7]—this is rare (estimated frequency < 0.03%). In this case, the overall degree of platelet count fall is not unexpected for a postcardiac surgery patient [7], likely reflecting the consequences of cardiac surgery and RCC transfusion (hemodilution), hemodynamic instability (increased platelet consumption), and the usual lag time of 2 to 3 days pending bone marrow response to acute perioperative thrombocytopenia [8].

**FIGURE 2:**
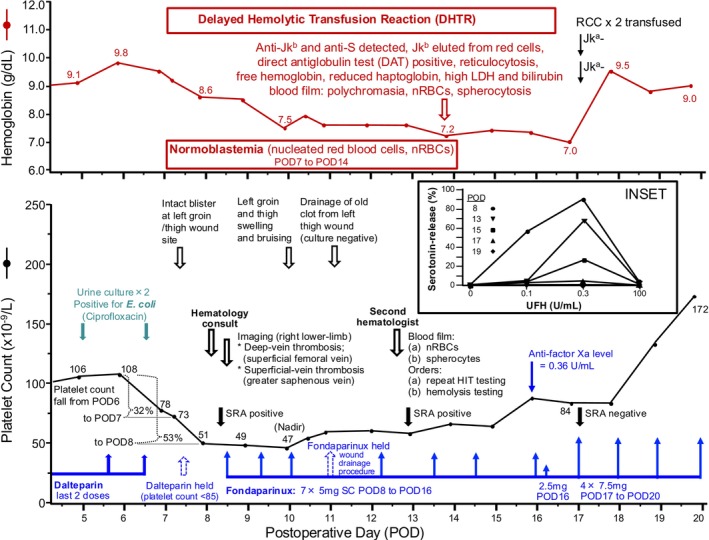
Serial platelet counts (lower panel) and hemoglobin levels (upper panel) from postoperative day (POD) 5 onwards. Platelet count and hemoglobin declines that both began on POD7 ultimately led to the diagnoses of heparin‐induced thrombocytopenia (HIT) and delayed hemolytic transfusion reaction (secondary to anti‐Jk^b^ and anti‐S alloantibodies). Recovery from HIT‐associated thrombocytopenia was delayed by 10 days (from platelet count nadir of 47 × 10^9^/L on POD10 until rise to greater than 150 × 10^9^/L on POD20). The inset shows results of the serotonin‐release assay (SRA) when testing serial serum samples (PODs 8, 13, 15, 17, and 19): The absence of reactivity at buffer control (i.e., 0% serotonin‐release by all tested sera at 0 U/mL heparin), and the rapid decline in SRA reactivity (seroreversion) from POD8 to POD17/POD19, argue against autoimmune HIT (aHIT) as an explanation for delayed platelet count recovery. HIT, heparin‐induced thrombocytopenia; LDH, lactate dehydrogenase; nRBCs, nucleated red blood cells; POD, postoperative day; RCC, red cell concentrate; SC, subcutaneous; U, units. [Color figure can be viewed at wileyonlinelibrary.com]

**After reaching the postoperative platelet count nadir on POD2, the platelet count progressively rose to a peak of 108 × 10^9^/L on POD6; however, the platelet count then declined by 32% to 73 × 10^9^/L (POD7) and subsequently by 53% to 51 × 10^9^/L (POD8; all percentages in relation to the peak platelet count of 108) (Figure** **2** **). The hemoglobin on POD8 was 8.6 g/dL (decrease by 9.8 g/dL from 2 days earlier) (Figure** **2** **), and the WBC count was markedly elevated at 20.5 × 10^9^/L (increase from 15.8 2 days earlier). The dalteparin was automatically held on POD7, due to hospital protocol to not administer LMWH post‐surgery for platelet counts less than 85 × 10^9^/L. Urine culture grew *E. coli*, and ciprofloxacin was started. Based on the further drop in platelet count on POD8, blood cultures were ordered, and a hematology consultation was requested. Physical examination showed evidence of a left groin/thigh wound hematoma (at previous line insertion site). The hematologist requested: (a) screening tests for disseminated intravascular coagulation (DIC), (b) testing for HIT antibodies, and (c) lower‐limb imaging for deep‐vein thrombosis (DVT). The DIC screen showed elevated d‐dimer of > 4000 μg/L FEU (RR, < 500), an activated partial thromboplastin time (APTT) of 27 s (RR, 22–35), a thrombin clotting time of 31 s (RR, 20–30), an international normalized ratio (INR) of 1.1 (RR, 0.8–1.2) and a fibrinogen level of 210 mg/dL (RR, 160–420). Duplex ultrasound showed non‐occlusive deep‐vein thrombosis (DVT) in the right lower limb (proximal superficial‐femoral vein) and superficial vein thrombosis (right greater saphenous vein). The hematologist calculated a 4Ts score as follows: 2 points for *T*hrombocytopenia (> 50% fall), 2 points for *T*iming (onset of thrombocytopenia, POD7), 2 points for *T*hrombosis (confirmed DVT) and 1 point for o*T*her possible causes of thrombocytopenia (possible infection‐associated thrombocytopenia), for a total score of 7 points (i.e., high pretest probability).**

HIT is an important clinical‐pathological disorder characterized by unexpected thrombocytopenia that bears a temporal relationship with a proximate immunizing exposure to heparin and an associated high risk for thrombosis (relative risk, 12‐15‐fold) [9]. HIT is caused by antibodies that recognize multimolecular complexes formed by the cationic protein, platelet factor 4 (PF4), when it binds to the polyanion, heparin. Pathogenic antibodies of IgG class form PF4‐heparin‐IgG complexes on platelet surfaces that activate platelets via their FcγIIa receptors. Diagnosis is ultimately based upon a compatible clinical picture and detection of pathogenic platelet‐activating HIT antibodies [9]. As results of lab testing for HIT antibodies can sometimes take one or more days, early treatment decisions are often made based upon pretest probability (PTP), e.g., per 4Ts score [10]. (This case occurred prior to the approval of any rapid immunoassays for HIT in Canada.) The 4Ts is a validated scoring system to assess the clinical presentation of HIT with a high negative predictive value (at least 98%). A score of 3 points or fewer indicates a low PTP, between 4 and 5 points indicates a moderate PTP, and between 6 and 8 indicates high PTP [10]. A 4Ts score of 7 points was assigned to this patient, with only 1 point for the last parameter based upon her elevated WBC count indicating possible sepsis (she had a documented urinary tract infection and also potentially infected left groin/thigh wound site). However, one study found that leukocytosis itself can occur in HIT, particularly in association with HIT‐associated thrombosis [11]. The increased d‐dimer level and relatively low fibrinogen level of 210 mg/dL (typically, fibrinogen levels are 450 mg/dL or higher several days following surgery or major trauma [12]) are also consistent with consumptive thrombocytopenia characteristic of HIT.

**Blood cultures were negative. Given a working diagnosis of acute HIT, but also concerns of recent wound hematoma, the hematologist ordered fondaparinux at an intermediate dose of 5 mg daily (weight = 70 kg; creatinine = 0.92 mg/dL [RR, 0.57–1.13]).**

Immediate management of HIT involves discontinuation of heparin and substitution of a non‐heparin anticoagulant, usually in therapeutic doses. Anticoagulant options include indirect (i.e., antithrombin‐dependent) factor Xa inhibitors (fondaparinux, danaparoid [not available in the U.S.]), direct Xa inhibitors (rivaroxaban, apixaban), and parenteral direct thrombin inhibitors (argatroban, bivalirudin) (Table 1) [13]. Platelet transfusions are usually avoided (theoretical risk of promoting thrombosis). In this hospital, fondaparinux is the most frequently prescribed anticoagulant to manage HIT due to ease of administration (subcutaneous, once‐daily), direct monitoring through anti‐factor Xa levels, and possibly lower bleeding complications versus DTIs [14]. Although fondaparinux itself has rarely been implicated in causing HIT [15], the risk of already existing HIT antibodies cross‐reacting with fondaparinux is low [16]. Efficacy (i.e., no thrombotic complications after starting fondaparinux) appears to be at least 90% [17]. Warfarin should not be administered until the patient's platelet count recovers (≥ 150 × 10^9^/L) as it may worsen thrombosis through reduced protein C synthesis [13].

**TABLE 1:** American Society of Hematology (ASH) guidelines for treatment of HIT in relation to U.S. and Canadian drug approvals.

Non‐heparin anticoagulant	Advice per ASH treatment guidelines	Approved by U.S. Food and Drug Administration (FDA) to treat HIT	Approved by health Canada to treat HIT
Indirect factor Xa inhibitor (parenteral) Danaparoid sodium Fondaparinux	✓^a^ ✓^a^, ^b^		✓
Direct thrombin inhibitor (parenteral)			
Argatroban	✓^a^, ^c^	✓	✓
Bivalirudin	✓^a^, ^c^	✓^d^	✓^d^
Direct oral anticoagulant (DOAC)	✓^a^, ^b^, ^e^		
Vitamin K antagonist (VKA)	X^f^	X^g^	X^g^

*Note:* Anticoagulants indicated with a check mark (✓) in the second column are suggested treatment options per the ASH 2018 treatment guidelines, as follows: “Recommendation 3.1: When a non‐heparin anticoagulant is being selected, the ASH guideline panel suggests argatroban, bivalirudin, danaparoid, fondaparinux, or a DOAC.” [13] In contrast, recommendation against use (contraindication) during the acute thrombocytopenic phase of HIT is indicated by an “X.”

^a^ Per ASH HIT treatment guidelines: “In patients with HIT complicated by life‐ or limb‐threatening thromboembolism (eg, massive pulmonary embolism or venous limb gangrene), a parenteral non‐heparin anticoagulant may be preferred, because few such patients have been treated with a DOAC.” [13].

^b^ Per ASH HIT treatment guidelines: “Fondaparinux and the DOACs are reasonable options in clinically stable patients at average risk of bleeding. The same contraindications to their use in the treatment of acute VTE should be applied in determining their appropriateness for patients with HIT.” [13].

^c^ Per ASH HIT treatment guidelines: “In patients with critical illness, increased bleeding risk, or increased potential need for urgent procedures, argatroban or bivalirudin may be preferred because of shorter duration of effect. These patients may require close monitoring. In patients with moderate or severe hepatic dysfunction (Child‐Pugh Class B or C), it is advisable to avoid argatroban or use a reduced dose.” [13].

^d^ Bivalirudin indication for HIT per FDA is only listed for patients with HIT undergoing percutaneous coronary intervention (PCI), rather than for the management of HIT more generally. For Health Canada, both PCI and heart surgery in the setting of HIT are listed as indications for bivalirudin (note: the authors do not recommend use of bivalirudin for cardiac surgery).

^e^ Per ASH HIT treatment guidelines: “With respect to the choice of DOAC, most of the published experience in HIT is with rivaroxaban. Various dosing regimens have been reported. For patients with acute HITT, rivaroxaban at a dose of 15 mg twice per day for 3 weeks followed by 20 mg once per day is preferred. For patients with acute isolated HIT, rivaroxaban 15 mg twice per day until platelet count recovery (usually a platelet count of ≥ 150 × 10^9^/L) followed by 20 mg once per day is preferred if there is an indication for ongoing anticoagulation.” [13].

^f^ “Recommendation 3:5: In patients with acute HITT or acute isolated HIT, the ASH guideline panel recommends against initiation of a VKA before platelet count recovery (usually a platelet count of ≥ 150 × 10^9^/L) (strong recommendation, moderate certainty in the evidence about effects). Remark: This recommendation also applies to patients who are taking a VKA at the onset of acute HITT or acute isolated HITT. In these patients, the VKA would be discontinued and intravenous vitamin K would be administered concomitant with initiation of a non‐heparin anticoagulant.” [13].

^g^ Per product monographs (for the USA and Canada), warfarin (VKA) is contraindicated for use during acute HIT.

**Fondaparinux was held on POD11 so the left groin/thigh wound site could be opened, examined, and drained; a large amount of old‐appearing blood was evacuated. The HIT test results (POD8 sample) returned back as follows: IgG‐specific PF4/heparin enzyme‐linked immunosorbent assay (ELISA) = 2.53 OD (optical density) units (RR < 0.45); serotonin‐release assay (SRA) = 89% release at 0.3 U/mL heparin (RR < 20%, i.e., strong‐positive). A second hematologist reviewed the case on POD13 and agreed with the working diagnosis of HIT, but was concerned about the persisting thrombocytopenia and the falling hemoglobin from 9.8 g/dL on POD6 to 7.2 g/dL on POD14 (Figure** 2 **[upper panel]). The second hematologist ordered repeat HIT testing (POD13 sample—also SRA‐positive, but POD17 sample SRA‐negative) and tests for hemolysis and DIC, and also an anti‐factor Xa fondaparinux level; ultimately, a low trough anti‐Xa level resulted in a dose increase in fondaparinux (from 5 to 7.5 mg once‐daily).**

A falling hemoglobin value in a postoperative patient always raises concern regarding bleeding, either from wound sites or bleeding potentially related to anticoagulation and antiplatelet therapy. Indeed, bleeding is a not uncommon complication of anticoagulant therapy for HIT [18]. Evaluation of anticoagulant drug levels can be appropriate. In this case, the apparent stable left groin/thigh hematoma (likely indicating early postoperative bleeding), as well as the clear onset of progressive anemia that began at POD7, also raise the issue of possible immune‐mediated hemolysis, for example, secondary to alloantibodies formed in relation to the 6 units of RBCs transfused, or perhaps to drug‐dependent immune hemolysis (e.g., ciprofloxacin).

**Peripheral blood examination showed polychromasia, normoblastemia, and spherocytosis. The absolute reticulocyte count was markedly elevated at 500 × 10^9^/L (RR, 10–86), with free serum hemoglobin and reduced haptoglobin; her direct antiglobulin test was positive (both for IgG and C3); elution studies showed anti‐Jk^b^ coating her red cells. Her serum contained both anti‐Jk^b^ and anti‐S alloantibodies. Total bilirubin levels were elevated (total bilirubin, 2.9 mg/dL [RR, 0.3–1.0]), with the majority (2.2 mg/dL) representing unconjugated bilirubin. Lactate dehydrogenase (LDH) level on POD16 was 840 U/L (RR, 100–220). Further history showed no remote transfusion history, but the patient had borne several children.**

Laboratory evidence for hemolysis, together with serum anti‐Jk^b^ and anti‐S alloantibodies, plus anti‐Jk^b^ eluted from the patient's RBCs, and the timing of onset of hemolysis beginning 5–7 days posttransfusion of 6 units of RCCs, indicate a diagnosis of delayed hemolytic transfusion reaction (DHTR) [19]. Generally, such patients have been previously exposed to the implicated RBC alloantigens through previous transfusion or pregnancy (the latter applicable to this patient), with subsequent transfusion resulting in an anamnestic response, sometimes resulting in brisk and even life‐threatening hemolysis, especially if several units of allogeneic blood are hemolyzed. DHTRs occur in approximately 1 in every 2500 blood transfusions [20], with the most frequently implicated antigens involving the Rh, Kidd, Duffy, Kell, and MNS blood group systems [19, 20]. Given that the frequency of Jk^b^ antigens (part of the Kidd blood group system) is about 75% in Caucasians, and S (part of the MNS blood group system) is approximately 55%, it is statistically likely that most of the 6 units of transfused blood were incompatible with the newly generated anti‐Jk^b^ and anti‐S alloantibodies, and hence hemolyzed [21].

As the original exposure may have occurred months or even years before the secondary exposure (e.g., remote pregnancy), the patient's antibody titers can decrease significantly, becoming undetectable to antibody screening (as occurred in this case). This phenomenon is known as “evanescence” (or “seroreversion”): approximately one quarter of RBC alloantibodies disappear after 1 month, and 60%–70% disappear after 5 years [22].

**Repeat HIT testing, performed in the same assay using serum obtained on PODs 13, 15, 17, and 19, showed progressively less serotonin release, with no sample showing heparin‐independent serotonin release (Figure** 2 **inset). Eventually, the platelet count began to recover, beginning on POD19. Fondaparinux cross‐reactivity was ruled out (data not shown).**

## 2 Discussion

Our patient had the unusual clinical picture of two distinct immune‐mediated adverse events related to exogenous products administered for cardiac surgery. First, she developed the drug reaction, HIT, as a result of UFH administered during CABG surgery followed by LMWH given post‐surgery, complicated by thrombosis. Second, she developed alloimmune hemolysis due to anti‐Jk^b^ and anti‐S antibodies, presenting as DHTR. Of note, both immune‐mediated events began at approximately the same time (POD7). Whereas HIT was recognized within 48 h of the platelet count fall, the recognition of hemolysis was delayed, in part because the falling hemoglobin was initially attributed to wound hematoma (although the blood appeared old and had likely accumulated there shortly after surgery). Given a frequency of HIT of approximately 0.5% (1/200) post‐cardiac surgery with LMWH thromboprophylaxis [6], and DHTR frequency of approximately 0.04% (1/2500) [20], the probability of coincidentally developing both HIT and DHTR would be only 1/500,000 cardiac surgery patients who receive RCCs, that is, at most, one such patient expected every year or two in the United States and Canada.

An interesting aspect of the case was the delayed platelet count recovery after initiating fondaparinux anticoagulation. One potential explanation is “autoimmune HIT” (aHIT), where strong HIT antibodies with heparin‐*independent* properties cause ongoing platelet activation [23]; however, this was ruled out (Figure 2 inset). A second potential explanation was fondaparinux cross‐reactivity—also ruled out serologically. A third possibility was sepsis; however, blood cultures were negative, and the patient's clinical course did not seem compatible with severe infection. A fourth explanation is thrombocytopenia related to the alloimmune hemolysis. It is known that decreased platelet production—with thrombocytopenia—can complicate marked hemolytic processes [24, 25]. The coincidence of two relatively dramatic immune‐mediated complications—HIT and DHTR—is most unexpected, so whether alloimmune hemolysis can explain the patient's platelet count course may require future observations from other salient patient cases.

## Consent

The patient provided written permission for participating in the study relating to HIT (institutional approval is not required for case reports in our medical community).

## Conflicts of Interest

Theodore E. Warkentin has provided consulting services to Instrumentation Laboratory (Werfen), Paradigm Pharmaceuticals, and Veralox, and has provided expert witness testimony relating to HIT and non‐HIT thrombocytopenia. Myriam Beshai and Nour Alhomsi declare no relevant conflicts of interest.

## Data Availability

All data generated or analyzed during this study are included in this published article.
